# Can surgeons trust AI? Perspectives on machine learning in surgery and the importance of eXplainable Artificial Intelligence (XAI)

**DOI:** 10.1007/s00423-025-03626-7

**Published:** 2025-01-28

**Authors:** Johanna M. Brandenburg, Beat P. Müller-Stich, Martin Wagner, Mihaela van der Schaar

**Affiliations:** 1 Department of General, Visceral and Transplantation Surgery, Heidelberg University Hospital, Heidelberg, Germany; 2 National Center for Tumor Diseases (NCT), Heidelberg, Germany; 3 University Digestive Healthcare Center Basel, Basel, Switzerland; 4 Department of Visceral, Thoracic and Vascular Surgery, University Hospital Carl Gustav Carus, Technische Universität Dresden, Fetscherstraße 74, 01307 Dresden, Germany; 5 National Center for Tumor Diseases (NCT/UCC), Dresden, Germany; 6 Centre for Tactile Internet with Human-in-the-Loop (CeTI), Technische Universität Dresden, Dresden, Germany; 7 University of Cambridge, Cambridge, UK; 8 Department of Electrical and Computer Engineering, University of California– Los Angeles, Los Angeles, CA USA; 9 Alan Turing Institute, London, UK; 10 Centre for Mathematical Imaging in Healthcare Machine Learning and Artificial Intelligence, Faculty of Mathematics, University of Cambridge, Wilberforce Road, Cambridge, CB3 0WA UK

**Keywords:** Artificial intelligence, Explainable artificial intelligence, Machine learning, Minimally invasive surgery

## Abstract

**Purpose:**

This brief report aims to summarize and discuss the methodologies of eXplainable Artificial Intelligence (XAI) and their potential applications in surgery.

**Methods:**

We briefly introduce explainability methods, including global and individual explanatory features, methods for imaging data and time series, as well as similarity classification, and unraveled rules and laws.

**Results:**

Given the increasing interest in artificial intelligence within the surgical field, we emphasize the critical importance of transparency and interpretability in the outputs of applied models.

**Conclusion:**

Transparency and interpretability are essential for the effective integration of AI models into clinical practice.

## Benefit or burden - how do surgeons perceive AI?

It is inarguable that there is immense potential in artificial intelligence (AI) and its most prominent subfield: machine learning (ML). This applies to surgery as well as to medicine in general, with healthcare being a highly data-generating and data-driven field. Experts in digital surgery [1] are convinced that emerging technologies can enhance preoperative planning, provide navigation assistance - similar to autonomous driving - by, e.g., highlighting the critical view of safety in the surgical field of minimally invasive procedures [2], assess surgical skills [3], and offer decision support, such as predicting intra- and postoperative complications [4] and personalizing therapy recommendations for complex patient cases.

Surgeons undergo extensive professional training, accompanied by high workload and stress levels. During years of collecting clinical experience, they gradually gain expertise regarding effective decision-making and managing situations with high demands on technical, cognitive, and communicative skills. This is especially important in the high-stakes environment of the operating room [5]. The potential integration of AI can thus justifiably evoke different reactions among the surgical community: doubt when it comes to a machine influencing what the correct patient diagnosis or treatment should be, e.g., in (surgical) oncology or the intensive care unit [6]; fear that at some point your technical qualities or ability of clinical reasoning will be replaced, e.g., in defining the critical view of safety in laparoscopic cholecystectomy [2]; or simply irritation because, e.g., automatic robot-assisted camera guidance may not precisely display the desired viewpoint.

## The importance of eXplainable artificial intelligence (XAI)

**Fig. 1:**
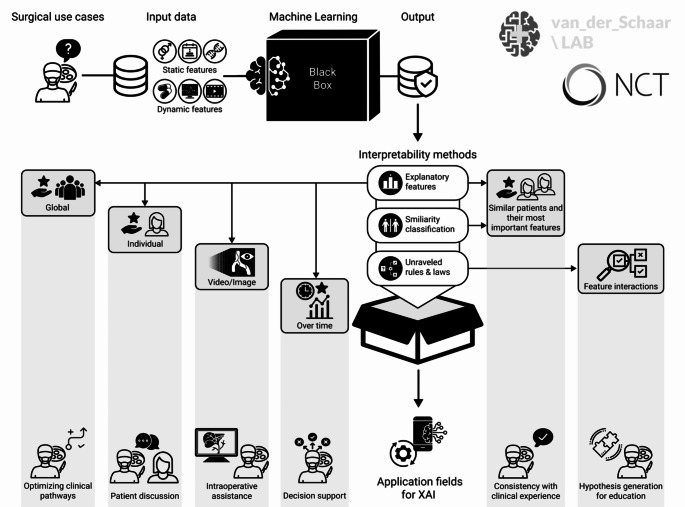
Methods and clinical applications for eXplainable artificial intelligence in surgery

Given the challenge of trust in AI systems and the limited concrete usage in healthcare, particularly in surgery [7], how can we foster the acceptance of ML methods and their integration into clinical practice? A major challenge is to make the ML models accessible to surgeons in such a way that the information is presented in a clear manner within an intuitive user interface, and their output being transparent and interpretable. Building trust and understanding is of utmost importance. As surgeons often find themselves in situations without much time to reconsider one’s actions or question recommendations, they need to be able to confidently rely on newly introduced AI-based assistance systems. This does not necessarily mean that the predictions of underlying ML models must be perfectly accurate, but rather that the surgeon understands why a certain output is generated by providing human-interpretable information including uncertainties and ambiguities that are inherent to clinical decision-making anyway. However, this is a major methodological challenge because the multi-layer architecture of neural networks used as ML models for high problem-complexity hardly allows humans to fully understand the models’ conclusions [8]. Researchers try to address this need by developing the field of ML model interpretability defined as the extent to which an ML model can be made understandable to relevant human users [9]. The term eXplainable AI (XAI) can thus be summarized as tailored interpretability for different users. It can be employed in many ways depending on the type of input data, the applied ML algorithm, and of course the requirements and questions of the users themselves [10]. To establish a surgical understanding of the capabilities of computer science in providing XAI, we present an overview of various interpretability methods with potential applications in surgery (Fig. 1).

### Global explanatory patient features

A ML model could predict the overall survival of patients undergoing different surgeries such as an esophagectomy based on preoperative, static patient data such as age, gender, comorbidities, neoadjuvant therapy, or smoker status. However, surgeons might need to know which data play the most important role for this prediction to optimize further treatment pathways. The interpretability method of explanatory (patient) features points out these features and their contribution to the model’s prediction.

### Individual explanatory patient features

However, the described global approach of outcome prediction in clinical practice is often not sufficient when it comes to specific patient cases with questions arising such as: “What are the most important features for the survival prediction of this specific patient I’m going to operate on tomorrow?”. The surgeon needs to know which subset of features is relevant for each patient meaning individualized feature importance [11], e.g., to build a basis for an informative patient discussion with specific recommendations.

### Explanatory features for imaging data

Image and video data are of particular importance in surgery, e.g., for surgical planning using radiological data or the surgical video itself during minimally invasive procedures. When these images are analyzed by means of computer vision, feature importance can be applied in the context of XAI by highlighting the parts of the image/video that have the greatest influence on the output when changed, so-called integrated gradients [12]. When developing a model to assess completeness of lymphadenectomy in esophagectomy, it is crucial for the surgeon to know exactly on which structures or features the model bases its rating in certain areas. In general, XAI may be more approachable for imaging tasks, as the visual nature of predictions often aligns with human interpretability, facilitating the detection and assessment of potential biases in the algorithm.

### Explanatory features taking time series into account

Especially in surgery with long and complex treatment pathways and the highly important surgical procedure itself, static features alone are insufficient for certain predictions. Interpretability approaches taking the temporal sequence of clinical processes into account [13] need to be addressed when, e.g., postoperative complications shall be predicted. Thereby, the risk estimation of developing anastomotic insufficiency after pancreatic surgery could be more effectively explained by assessing dynamic data such as intraoperative variation in heart rate and blood pressure of the patient during surgery, as well as the perioperative trends in laboratory values. Moreover, e.g., continuous monitoring of blood levels might enable conclusions about total blood loss, potential complications like vascular injuries, and correlations with specific surgical phases and steps. By incorporating temporal data, clinicians gain a deeper understanding of physiological patterns and their relationship to surgical outcomes.

### Similarity classification

In clinical practice, physicians learn a lot from comparing different but similar patient cases to each other, e.g., to find out whether their proposed intervention for the current patient is consistent with treated patients in the past. The interpretability method of similarity classification could derive similar patient cases when surgeons, e.g., must decide whether the risk of post hepatectomy liver failure after extended hemihepatectomy does or does not outweigh the potential benefits. Even more important may be information on the certainty a model has in its treatment recommendation for specific patients. XAI should thus further enable highlighting cases for which the model’s prediction is uncertain, supporting the surgeon’s right to be skeptical. Similarity classification could also help in this case, e.g., by being combined with individual explanatory patient features (see above). In this approach, similar patients and their most important features for the prediction for the actual patient are selected while showing if the model’s prediction for similar patients has been true or false [14]. Based on this, the surgeon has more information on whether to follow the model’s recommendation.

### Unraveled rules and laws

When surgeons prepare themselves for future patients or revisit a specific patient case, “what if” scenarios often arise: “What if the tumor had not been so close to the aorta? What if the patient had been 5 years younger? Under these circumstances, might a different intervention have been recommended, or could the patient have avoided a postoperative complication?”. These questions highlight the inherent complexity and variability in surgical decision-making. Scientists try to address these questions by uncovering previously unrevealed “rules” and “laws” in their models. By leveraging advanced analytical techniques, they seek to identify variable interactions that enhance our understanding of complex clinical scenarios. This process enables hypothesis generation offering insights into alternative pathways and potential outcomes for diverse patient scenarios.

### Surgeons should embrace eXplainable AI (XAI)

If assistance systems based on AI are to become an integral part of clinical care especially in surgery, explainability and the utilization of XAI are inevitable. While AI systems are not infallible and require continuous validation and optimization, one significant challenge lies in the inherent difficulty of explaining and comprehending the processes leading to a model’s output. XAI plays a crucial role in addressing this challenge, enabling surgeons to provide optimal care to their patients with the support of AI-based assistance systems in their final decisions. Simultaneously, incorporating XAI can simplify the process for users to articulate their doubts about specific ML-based recommendations and provide feedback to the model, thereby fostering a continuous building of trust. Thus, ML interpretability embedded in decision-support systems should be able to learn which interpretability modules are the most important and trust-worthy ones for individual clinicians, paving the way for personalized usage [9]. Concerning the challenge of developing an intuitive interface for digital AI tools in surgery, the emerging field of Large Language Models and Visual Language Models must be highlighted. With their ability to process and generate human-interpretable textual data, they could enable the integration of multimodal information, thereby facilitating the interaction of surgeons with conventional AI and explainability tools [15]. In terms of the rapidly developing field of artificial intelligence, surgeons should embrace XAI in surgery. Interpretable models need to be developed with methods taking the complex, dynamic and individual surgical patient cases into account and adapting to the user’s knowledge. This, however, is only possible if surgeons and computer scientists interact closely to bridge the gap between clinical need and technological feasibility.

## Data Availability

No datasets were generated or analysed during the current study.
